# Controlled Synthesis and Enhanced Photocatalytic Performance of MgAl_2_O_4_ Spinel Nanopowders for Environmental Applications

**DOI:** 10.1002/open.70263

**Published:** 2026-07-20

**Authors:** Yousra Taoudi, Hicham Oudghiri Hassani, Souad Rakass, Brahim El Bali, Mohammed Lachkar, Khalil Azzaoui, Belkheir Hammouti, Lamy Mamdoh Mohamed Hamed, Ahmed El‐Harairy

**Affiliations:** ^1^ Engineering Laboratory of Organometallic Molecular Materials and Environment (LIMOME) Faculty of Sciences Chemistry Department Sidi Mohamed Ben Abdellah University Fez Morocco; ^2^ Laboratory of Applied Organic Chemistry (LCOA) Chemistry Department Faculty of Sciences and Techniques Sidi Mohamed Ben Abdellah University Fez Morocco; ^3^ Euro‐Mediterranean University of Fes Fes Morocco; ^4^ Department of Environment and Agricultural Natural Resources College of Agricultural and Food Sciences King Faisal University Al‐Ahsa Eastern Province Saudi Arabia; ^5^ Department of Chemical and Biomolecular Engineering College of Engineering University of Nebraska–Lincoln Lincoln Nebraska USA; ^6^ Department of Soils and Water Faculty of Agriculture Damietta University Damietta Egypt

**Keywords:** magnesium aluminate spinel, nanopowders, photocatalytic degradation, Rhodamine B, Xenon lamp irradiation

## Abstract

Nanopowders of magnesium aluminate spinel (MgAl_2_O_4_) were produced using a solid‐state approach, involving a reaction between magnesium nitrate, aluminum nitrate, and oxalic acid. The synthesized nanomaterials’ structural, morphological, and catalytic properties were examined by XRD, FTIR, TGA, SEM, EDX, BET, and TEM analyses. To evaluate their catalytic performance, the photocatalytic degradation of Rhodamine B (RhB) dye under Xenon lamp irradiation was investigated. Therefore, the combined application of photocatalysis and adsorption processes has proven to be highly effective for removing RhB from aqueous solutions. Notably, the highest removal efficiency, reaching 95% after 120 min, was obtained through photocatalytic degradation under Xe lamp irradiation.

## Graphical abstract

A simple solid‐state route was employed to synthesize MgAl_2_O_4_ spinel nanopowders with high photocatalytic efficiency. The obtained nanomaterial achieved 95% degradation of Rhodamine B under Xe lamp irradiation, demonstrating its potential as an efficient photocatalyst for wastewater purification.

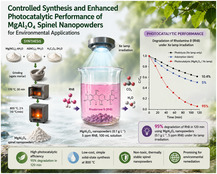

## 1 Introduction

Spinel‐type compounds are ternary oxides with the general formula AB_2_O_4_, where A and B are bivalent and trivalent ions. They crystallize in the cubic crystal system with space group $\text{Fd}\bar{3}m$ [1]. The A^2+^ cations occupy tetrahedral sites, while the trivalent B^3+^ ions are octahedrally coordinated sites [2]. Spinel‐structured materials are highly functional and extensively employed in various applications, such as refractory materials, sensors, high‐temperature components, lasers, electronics, and catalysis [3, 6]. Particularly, magnesium aluminate spinel (MgAl_2_O_4_) has attracted considerable attention from both academia and industry, owing to its thermal stability (melting point of 2135 °C), remarkable hardness, and strong mechanical resistance [7, 8]. Due to these properties, it has significant potential for high‐temperature and structural applications. For industrial applications such as adsorbents, catalysts, and catalyst supports, MgAl_2_O_4_ must meet several critical requirements, including high purity, nanoscale particle sizes, high surface area, and homogeneous pore structure [9, 11]. These characteristics are strongly influenced by the synthesis method employed. In fact, the title compound has been synthesized using various techniques, such as sol–gel processing [12, 13], solid‐state reactions [14, 15], Pechini method [16], hydrothermal method [17, 18], and coprecipitation [19, 22]. Each method offers distinct advantages regarding particle morphology, crystallinity, and surface characteristics. In addition to its synthesis methods, the structural and morphological diversity of MgAl_2_O_4_, resulting from a variety of preparation techniques, has enabled its use in a wide range of applications, including catalyst supports [23, 25], membranes [26, 28], adsorption [29, 31], and humidity sensors [32, 34]. Moreover, certain spinel materials, such as MAl_2_O_4_(M = Mg, Sr, Ba), and NiFe_2_O_4_, exhibit semiconducting properties, thereby enabling their use in photocatalytic processes such as pollutant degradation and water splitting [35, 36]. With growing interest in photocatalysis, particularly for environmental remediation, MgAl_2_O_4_ has recently drawn attention as a potential photocatalyst [37]. In fact, photocatalysis using semiconductor materials is regarded as an effective strategy to address global energy and environmental challenges [38, 41]. Under light irradiation, photocatalysts generate photo‐induced electron–hole pairs, which can initiate redox reactions for pollutant removal [42, 43]. However, to enable the large‐scale and economically viable implementation of photocatalytic technology in industry, the development of low‐cost and efficient photocatalysts remains essential. In this context, magnesium aluminate spinel (MgAl_2_O_4_) has emerged as a promising material. Its photocatalytic performance is typically assessed via the degradation of methylene blue under UV–visible light irradiation [44]. MgAl_2_O_4_ spinel was selected due to its high thermal and chemical stability and nontoxicity, which make it a promising material for photocatalytic applications. In particular, its potential in the degradation of organic dyes such as Rhodamine B (RhB) has attracted increasing attention. Indeed, RhB is a toxic and persistent organic pollutant that can cause serious environmental contamination due to its high chemical stability, poor biodegradability, and tendency to accumulate in aquatic ecosystems. Moreover, exposure to rhodamine dyes has been reported to be harmful to human health, with potential carcinogenic and mutagenic effects. Therefore, the efficient removal and photocatalytic degradation of such dyes is of significant importance for environmental protection and wastewater treatment [45, 46].

In this study, the resulting spinel‐type oxide nanopowders were prepared and extensively characterized using Fourier transform infrared spectroscopy (FTIR), thermogravimetric analysis (TGA), X‐ray diffraction (XRD), scanning electron microscopy (SEM), energy‐dispersive X‐ray spectroscopy (EDX), Brunauer–Emmett–Teller method (BET), and transmission electron microscopy (TEM). In addition, their adsorption capacity and photocatalytic activity were evaluated through the degradation of RhB dye under Xenon lamp irradiation.

## 2 Experimental Section

### 2.1 Synthesis of MgAl_2_O_4_ Nanoparticles

**FIGURE 1:**
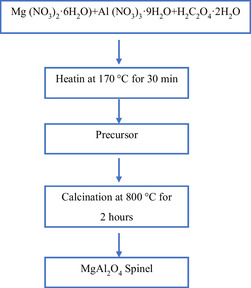
A schematic diagram of the preparation of the MgAl_2_O_4_ nanoparticles.

Powder of the spinel MgAl_2_O_4_ was synthesized via a solid‐state route that involved forming an oxalate precursor, as reported in our previous research [47, 49]. Concretely, magnesium nitrate (Mg (NO_3_)_2_·6H_2_O), aluminum nitrate (Al (NO_3_)_3_·9H_2_O), and oxalic acid (H_2_C_2_O_4_·2H_2_O) were mixed in the molar ratio of 1/2/6. The resulting mixture was thoroughly ground using an agate mortar to ensure homogeneity. The homogenized powder was then heated at 170 °C for 30 min, followed by a calcination at 800 °C for 2 h with a heating rate of 10 °C/min, to obtain magnesium aluminate spinel (MgAl_2_O_4_). A schematic diagram summarizing the methodology used for the preparation of MgAl_2_O_4_ nanoparticles is shown in Figure 1.

### 2.2 Characterizations

TGA was carried out using a LINSEIS STA PT1600 thermal analyzer. FTIR spectra were recorded with a BRUKER INVENIO‐S spectrometer over the 4000–400 cm^−1^ range. XRD measurements were performed on a PANalytical powder diffractometer equipped with Cu‐Kα radiation (*λ* = 1.54060 Å), operated at 40 kV and 30 mA. Diffraction patterns were collected within a 2*θ*‐range 10°–80°. Phase identification was carried out by comparing the obtained diffraction patterns with standard data from the Joint Committee on Powder Diffraction Standards (JCPDS) database. The average crystallite size (*D*) was calculated using the Scherrer equation:

D=kλ/(FWHM×cos θ)

where *k* = 0.9 is the shape factor, *θ* is the Bragg angle, and FWHM represents the full width at half maximum of the diffraction peak. The surface morphology was observed by SEM using a JEOL JSM‐IT500 HR system. Elemental analysis was performed through EDX integrated into the same SEM instrument, and TEM was performed using a JEOL 1400 microscope. The specific surface area, pore size distribution, and pore volume were determined using a Tristar II Plus. The specific surface area was calculated according to the BET method, while the average pore diameter and total pore volume were determined using the Barrett–Joyner–Halenda (BJH) model.

A UV–vis spectrophotometer (Camspec M550, double‐beam scanning) was used to monitor the degradation of RhB spectroscopically.

### 2.3 Photocatalysis Experiments

Using RhB photodegradation as a test reaction, the photocatalytic efficiency of MgAl_2_O_4_ nanocatalysts under xenon lamp irradiation was investigated. For the experiment, 0.1 g of MgAl_2_O_4_ nanostructures was added to 100 mL of RhB solution with a concentration of 5 ppm, and then dispersed by stirring for 30 min in the dark to establish an adsorption–desorption equilibrium between the dye molecules and the surface of the MgAl_2_O_4_ nanocatalyst. The mixture was then placed in a specially designed glass photoreactor and irradiated with a Xenon lamp. A 500 W Xenon lamp was positioned at the top of the photoreactor, at a distance of 10 cm from the reaction vessel. The photocatalytic tests were carried out at room temperature. At selected time intervals, a volume of 1.5 mL of the solution was collected through a microfilter‐equipped syringe and analyzed at 554 nm using a Camspec M550 UV–visible spectrophotometer, where RhB shows maximum absorbance.

## 3 Results and Discussion

### 3.1 Characterizations of the Magnesium Aluminate Complex

**FIGURE 2:**
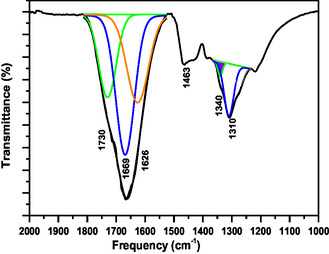
Deconvolution of the infrared (IR) spectrum of MgAl_2_O_4_ complex.

Figure 2 presents the FTIR spectrum of the magnesium aluminate complex, displaying a single broad absorption band in the 1800–1550 cm^−1^ region. Deconvolution of this band reveals the presence of several distinct components. Specifically, the absorption bands observed between 1730 and 1669 cm^−1^ are attributed to the C=O stretching vibrations of the oxalate group [38, 41, 50, 51]. This corresponds well with the C—O stretching vibration detected at 1463 cm^−1^. Also, the bands around 1310 and 1340 cm^−1^ correspond respectively to the δ(OCO) bending vibration and the υ(C—O) stretching vibration [52, 53]. Finally, the absorption band at 1626 cm^−1^ is assigned to the bending vibration δ(H_2_O), confirming the presence of water molecules [54].

**FIGURE 3:**
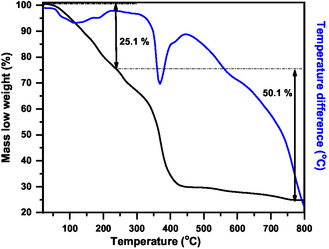
TGA of MgAl_2_O_4_ complex.

To further analyze the prepared complex, TGA and differential thermogravimetric analysis (DTG) were performed. We depict the related results in Figure 3. The thermogram reveals that the thermal decomposition proceeds in two distinct stages. The first stage, occurring between 25 and 240 °C, exhibits a gradual weight loss of 25.1%, which is attributed to the removal of water molecules from the precursor. The second stage, between 240 and 800 °C, with 50.1%, results from the decomposition of the oxalate precursor.

Based on the combined FTIR and TGA data analyses, as well as on the possible oxidation states of magnesium and aluminate, we propose the chemical formula MgAl_2_(C_2_O_4_)_4_·8H_2_O. Up to 800 °C, the experimental weight of 24.80% is close to the theoretical prediction of 24.73% for the expected composition.

### 3.2 Magnesium Aluminate Characterization

#### 3.2.1 XRD Analysis

**FIGURE 4:**
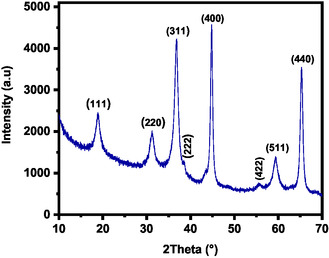
XRD pattern of nanopowder MgAl_2_O_4_ obtained at 800 °C.

The XRD pattern presented in Figure 4 corresponds to a representative sample of MgAl_2_O_4_ calcined at 800 °C. The pattern reveals a single cubic phase with space group Fd‐3m, consistent with the standard JCPDS card No. 01‐084‐0377 for MgAl_2_O_4_. The lattice parameters for this phase are *a* = b = *c* = 8.0831 Å and *α* = *β* = *γ* = 90°. The XRD analysis confirms the absence of any impurity phases [55]. The diffraction peak at 2*θ* = 18.8°, corresponding to the (111) plane and the highest d‐spacing, was used to determine the crystallite size (*D*), which was found to be 24 nm.

#### 3.2.2 Optical Properties

The photonic energy (*E*_g_) of MgAl_2_O_4_ nanoparticle has been calculated using Tuac's equation (*E*_g_) (Equation (1)):

(1) $${\left(\alpha h\nu \right)}^{n}=B(h\nu ‐E_{g})$$

where *α* is the absorption coefficient, *B* is a constant, *hν* is the energy of the incident photon, *E*_g_ is the optical bandgap, and *n* is an index that takes values such as 2, 3, 1/2 or 1/3, depending on the type of band‐to‐band transitions.

**FIGURE 5:**
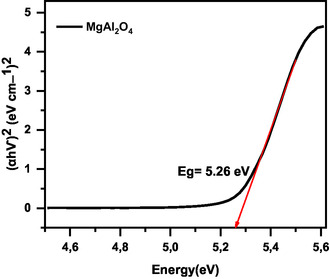
Tauc plots for determining the optical bandgap of MgAl_2_O_4_ nanoparticles calcined at 800 °C for 2 h.

Accordingly, the bandgap energy of the MgAl_2_O_4_ nanoparticles was determined using the Tauc method by plotting the function (*αhν*)^2^ as a function of photon energy (*hν*). Then, the linear portion of the absorption edge was extrapolated along the energy axis to estimate the bandgap. As shown in Figure 5, the optical bandgap of MgAl_2_O_4_ spinel nanoparticles was determined, and the direct bandgap was estimated to be 5.26 eV.

In addition, the reduction in bandgap energy can be attributed to the accumulation of defect states between the valence and conduction bands. Consequently, MgAl_2_O_4_ spinel nanoparticles can be considered promising materials for applications in the fields of semiconductors and photocatalysis [56, 57].

#### 3.2.3 Scanning Electron Microscope, Elemental Mapping, and EDX Analysis

**FIGURE 6:**
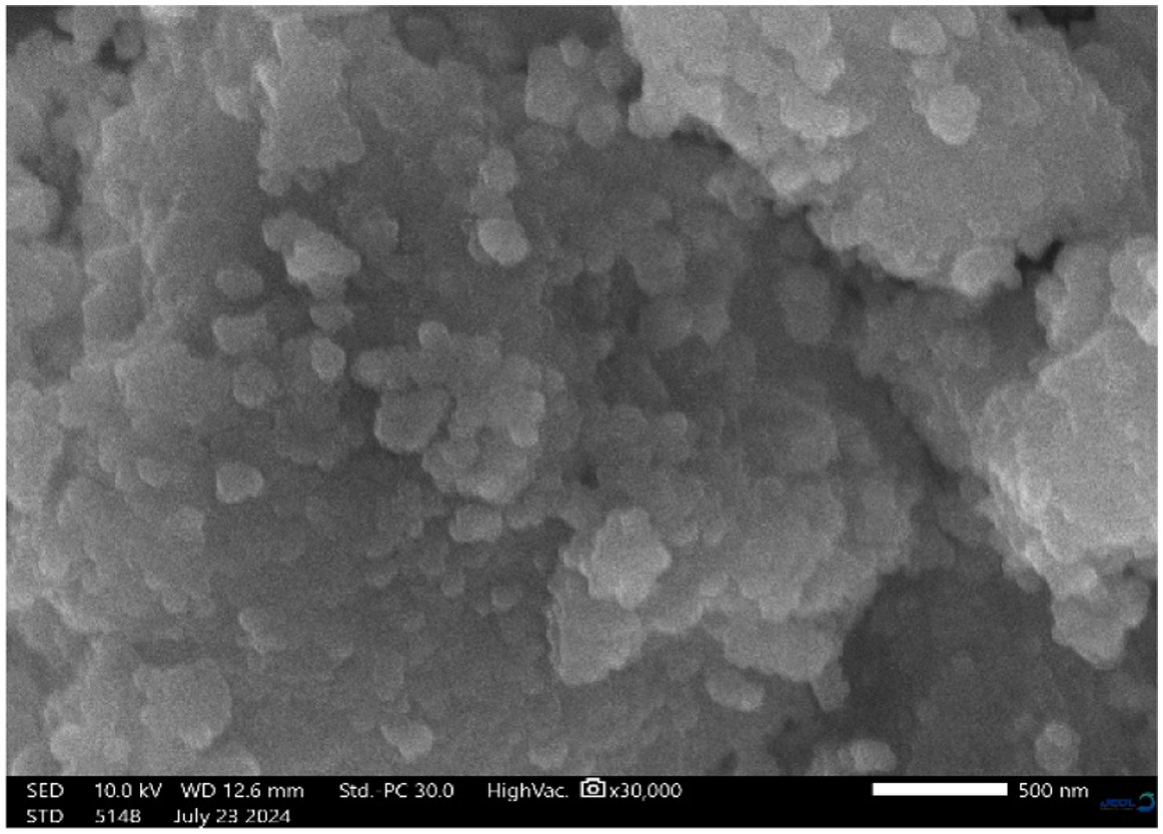
SEM micrographs of MgAl_2_O_4_ nanoparticles.

The surface morphology of MgAl_2_O_4_ was investigated using SEM, and the corresponding micrographs are presented in Figure 6. The particles exhibit a spherical morphology with remarkable aggregation. The agglomerates appear to be formed by the accumulation of smaller particles, with sizes ranging from 50 to 107 nm. Additionally, the particles display a predominantly spherical shape.

**FIGURE 7:**
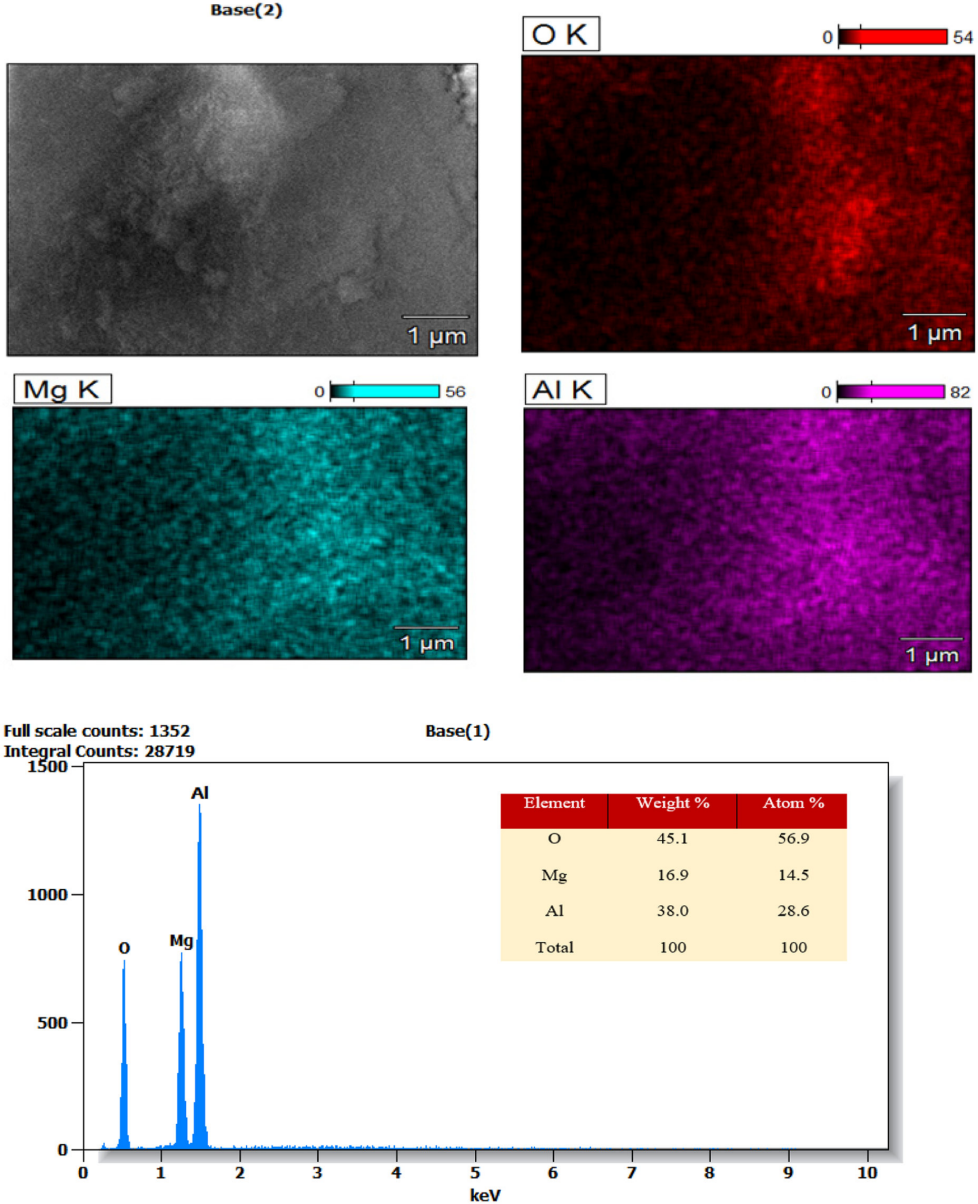
EDX spectrum and elemental mapping of MgAl_2_O_4_ synthesized at 800 °C.

Figure 7 presents the elemental mapping of MgAl_2_O_4_, indicating a homogeneous spatial distribution of magnesium (Mg), aluminum (Al), and oxygen (O) across the particle structure. The corresponding EDX spectrum analysis confirms the presence of the constituent elements of the synthesized phase. The atomic percentages were determined to be 56.9% for O, 14.5% for Mg, and 28.6% for Al, in good agreement with the expected stoichiometry of MgAl_2_O_4_.

**FIGURE 8:**
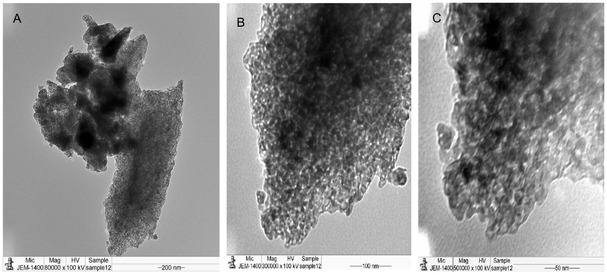
TEM micrographs of MgAl_2_O_4_ nanoparticles at three different magnifications; MgAl_2_O_4_ (A) ×80,000, (B) ×300,000, and (C) 500,000.

The TEM image of the spinel MgAl_2_O_4_ powder (Figure 8A–C) reveals that the MgAl_2_O_4_ nanoparticles exhibit a spherical‐like morphology, with an average particle size of less than 50 nm (in good agreement with the XRD results) The smaller value (24 nm) obtained from XRD can be explained by the fact that this technique determines the crystallite size and not the particle size, whereas SEM and TEM reflect the size of aggregated particles, ranging from 50 to 107 nm. The agglomeration of crystallites thus leads to the formation of larger particles.

**FIGURE 9:**
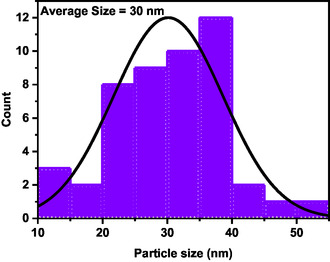
Particle size distribution histogram of MgAl_2_O_4_ for the corresponding TEM images.

The particle size distribution histogram obtained from the corresponding TEM images is shown in Figure 9, demonstrating that the MgAl_2_O_4_ nanoparticles are relatively well dispersed, with a particle size distribution centered around a mean value of approximately 30 nm and a standard deviation of 13.85 nm. This indicates moderate dispersion of particle sizes. The average sizes measured from TEM images and calculated from XRD patterns generally agree well.

#### 3.2.4 Surface Area Analysis

**FIGURE 10:**
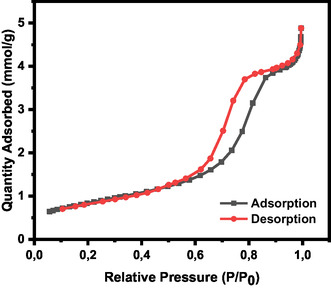
Adsorption and desorption curves obtained from the BET measurements of the MgAl_2_O_4_ nanoparticles.

The synthesized Magnesium aluminate spinel (MgAl_2_O_4_) has a BET surface area of 67.73 m^2^/g, which corresponds to a particle size of 24.74 nm calculated using the equation *D*_BET_ = 6000/*d*.*S*, where *d* is the density (3.58 g/cm^3^), and *S* is the specific surface area. It is a similar value to that calculated from the XRD pattern. On the other hand, as depicted in Figure 10, the adsorption/desorption isotherms are type IV, exhibited by mesoporous solids, and exhibit the characteristic H1 hysteresis loop (IUPAC classification), which is consistent with mesoporous or nanoporous materials [58]. The pores are cylindrical or slit‐shaped. The pore size was found to be 10.2 nm, and the total pore volume was found to be 0.1695 cm^3^/g using the BJH method.

#### 3.2.5 Photolysis, Adsorption, and Photocatalysis of RhB Dye Using MgAl_2_O_4_

**FIGURE 11:**
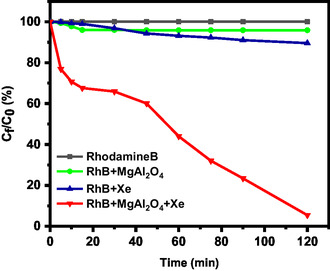
Adsorption and photodegradation of RhB dye over MgAl_2_O_4_ catalysts under Xe lamp irradiation.

The photodegradation of RhB by xenon lamp alone (photolysis), in the presence of MgAl_2_O_4_ under Xe lamp irradiation (photocatalysis), and in the absence of xenon lamp with MgAl_2_O_4_ (adsorption) is shown in Figure 11. It is clearly demonstrated that in the presence of MgAl_2_O_4_ without a light source (adsorption condition), only a limited decrease of approximately 5% in dye concentration was observed. It is important to note that the sample was kept in the dark for 120 min to allow the adsorption–desorption equilibrium to be established. In the absence of the MgAl_2_O_4_ catalyst (i.e., under photolysis conditions), the degradation efficiency of RhB reached only 10.4% after 120 min of Xe lamp irradiation. However, when MgAl_2_O_4_ was added as a photocatalyst (i.e., under photocatalytic conditions), the degradation efficiency increased to 95% under the same irradiation conditions and time interval (120 min). This result demonstrates the high catalytic efficiency of the synthesized MgAl_2_O_4_ in the photodegradation of RhB by xenon lamp irradiation. A comparison to previous research works found in the literature is presented in Table 1.

**TABLE 1:** Comparative analysis of reaction times and degradation percentages of RhB dye using MgAl_2_O_4_ photocatalysts.

Samples	Type	Dye	Lamp	***C***_**dye**_ **, mgL** ^**−1**^	***C***_**catalyst**_ **, gL** ^**−1**^	Degradation, %	Reaction time, min	Reference
MgAl_2_O_4_	Nanoparticles	RhB	mercury lamp	10	0.1	80	140	[59]
MgAl_2_O_4_:Ce	Composite	RhB	Xenon lamp	5	1.5	88.2	180	[60]
MgAl_2_O_4_	Nanocrystalline	RhB	Sunlight	10	0,01	68	150	[61]
MgAl_2_O_4_	Nanoparticles	RhB	Xe lamp	5	0.1	95	120	This work

**FIGURE 12:**
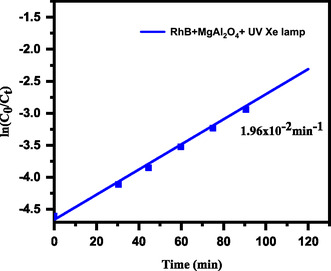
Kinetic curve of RhB degradation under Xe lamp irradiation in the presence of MgAl_2_O_4_ nanocatalysts.

The kinetics of the photocatalytic degradation of RhB on MgAl_2_O_4_ nanoparticles were evaluated using the pseudo‐first‐order kinetic model based on the Langmuir–Hinshelwood approach. The kinetic equation can be described as follows:

$$Ln(C_{0}/C_{t})=kt$$

where (*k*) is the apparent first‐order rate constant (min^−1^), (*C*_0_) is the initial concentration of RhB, and (*C* *_t_*) is the concentration at irradiation time (*t*). As shown in Figure 12, a good linear relationship was obtained from the plot of Ln (*C*_0_/*C* *_t_*) versus irradiation time, confirming that the photocatalytic degradation of RhB follows pseudo‐first‐order kinetics. The apparent rate constant was calculated to be 1.96 × 10^−2^ min^−1^. This result indicates the efficient photocatalytic activity of the MgAl_2_O_4_ nanocatalyst under Xe lamp irradiation.

**FIGURE 13:**
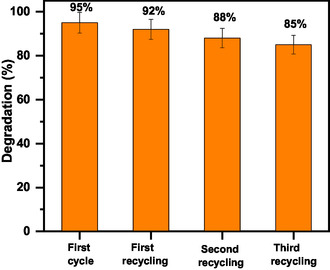
Recycled catalyst against degradation of RhB under Xe lamp irradiation in the presence of MgAl_2_O_4_ nanocatalysts (*C* = 5 ppm, *m* = 0.1 g, and Time = 120 min).

On the other hand, the recyclability of the prepared photocatalyst was investigated by performing three successive degradation cycles of RhB under identical operating conditions. As illustrated in Figure 13, the photocatalytic performance gradually declined, decreasing from nearly 95% during the first cycle to about 85% after the third cycle. Such a reduction in activity can be attributed to several factors commonly observed in heterogeneous photocatalytic processes. First, a minor loss of catalyst during the separation and cleaning procedures may lead to a decrease in the number of accessible active sites. Second, the deposition of intermediate degradation products on the catalyst surface can impede the adsorption of RhB molecules by covering the active sites. Furthermore, repeated use may induce slight changes in the structural and surface properties of the photocatalyst, thereby contributing to the reduction in its activity. Nevertheless, the catalyst still maintained a relatively high degradation efficiency of approximately 85% after three cycles, demonstrating its satisfactory stability and good potential for repeated applications.

#### 3.2.6 Photocatalysis Mechanism

**FIGURE 14:**
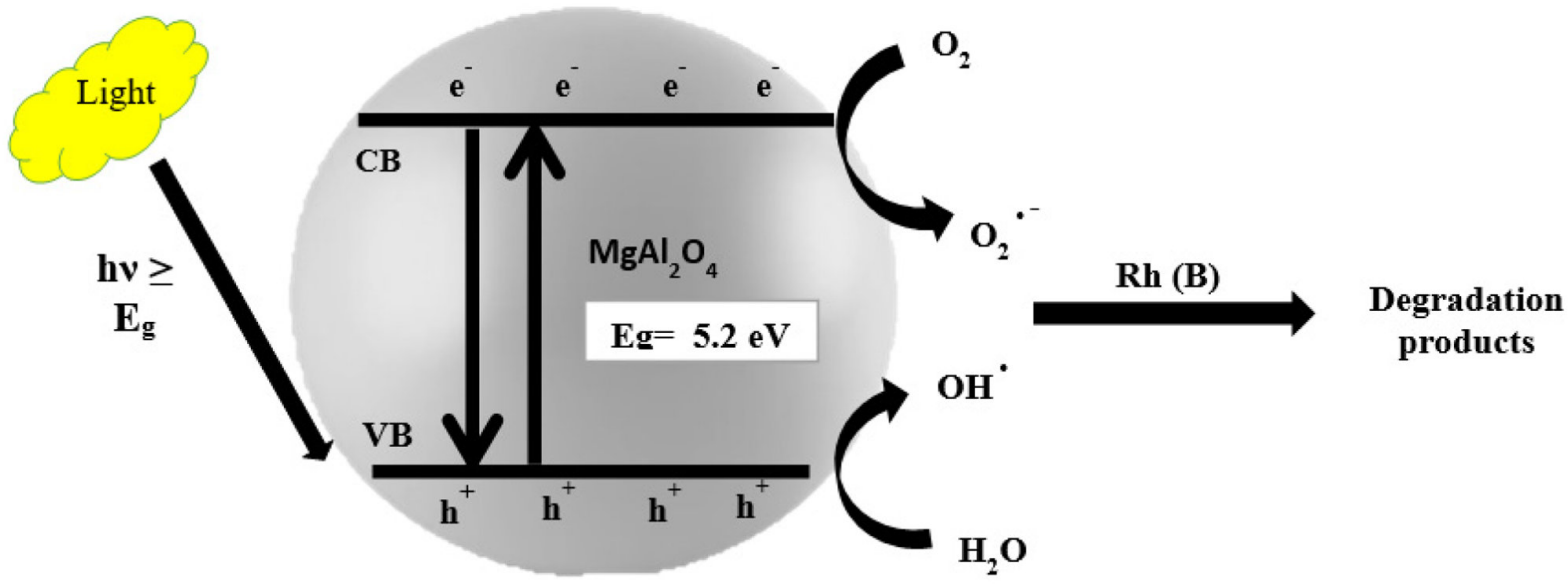
Reaction mechanism of RhB photodegradation over MgAl_2_O_4_ nanoparticles under Xe lamp irradiation.

A proposed mechanism for this photocatalytic reaction can be proposed as follows. Upon UV irradiation of the RhB solution containing the catalyst, electrons are excited from the valence band to the conduction band, resulting in the generation of electron–hole (*e* ^−^/*h* ^+^) pairs. These charge carriers play a crucial role in the formation of reactive radicals. The photogenerated holes and electrons react with H_2_O and dissolved O_2_, respectively, leading to the formation of hydroxyl radicals (OH*) and superoxide radicals (O_2_*). These highly reactive species subsequently attack the RhB molecules adsorbed on the surface of the MgAl_2_O_4_ catalyst, leading to their degradation into simpler and less harmful products [62, 66]. The probable mechanism of the photocatalytic degradation of RhB can be summarized by the following reactions and is schematically illustrated in Figure 14:

$${\mathrm{MgAl}}_{2}O_{4}+h\nu \;\to \;{\mathrm{MgAl}}_{2}O_{4}+e^{‐}+h^{+}$$

$$h^{+}+H_{2}O\;\to \;{\mathrm{OH}}^{*}$$

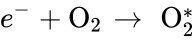







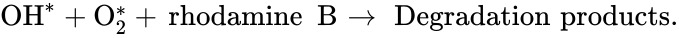




Similar degradation pathways of organic dyes over MgAl_2_O_4_‐based photocatalysts have been reported in previous studies [37, 66, 67].

## 4 Conclusion

We successfully synthesized MgAl_2_O_4_ nanoparticles using a solid‐state method at 800 °C. According to XRD, SEM, and EDX analyses, the MgAl_2_O_4_ nanoparticles prepared in this way exhibit high purity and a cubic crystal structure known as a spinel structure, with homogeneous shapes and sizes ranging from 24 to 187 nm. When the as‐prepared nanoparticles were used as a photocatalyst, approximately 95% degradation of RhB was achieved after 120 min of Xenon lamp irradiation. MgAl_2_O_4_ is a promising material, with high potential for photocatalytic applications under Xe lamp irradiation.

## Author Contributions

**Yousra Taoudi**: writing – original draft, writing – review & editing, methodology, formal analysis, investigation, conceptualization. **Hicham Oudghiri Hassani**: software, investigation, methodology, project administration, validation, writing – review & editing, supervision. **Souad Rakass**: data curation, software, investigation. **Brahim El Bali**: software, formal analysis. **Mohammed Lachkar**: supervision. **Khalil Azzaoui**: writing – review & editing, validation, project administration, resources. **Belkheir Hammouti**: formal analysis, validation. **Lamy Mamdoh Mohamed Hamed**, and **Ahmed El‐Harairy**: methodology, formal analysis, writing – review & editing, supervision.

## Funding

This study was supported by Deanship of Scientific Research, Vice Presidency for Graduate Studies and Scientific Research, King Faisal University, Saudi Arabia (KFU263633).

## Conflicts of Interest

The authors declare no conflicts of interest.

## Data Availability

Comprehensive and explicit characterizations of the utilized materials and instruments are articulated within the materials and methods segment of the manuscript. Furthermore, the acquired data is substantiated through the citation of the figures and tables present in the manuscript. Overall, all data that were produced or examined throughout this investigation are encompassed within this published article.
